# GCNTrack: A Pig-Tracking Method Based on Skeleton Feature Similarity

**DOI:** 10.3390/ani15071040

**Published:** 2025-04-03

**Authors:** Zhaoyang Yin, Zehua Wang, Junhua Ye, Suyin Zhou, Aijun Xu

**Affiliations:** 1 School of Mathematics and Computer Science, Zhejiang Agriculture and Forestry University, Hangzhou 311300, China; y145056@163.com (Z.Y.); relaxwang0714@gmail.com (Z.W.); 2 School of Environmental and Resource Science, Zhejiang Agriculture and Forestry University, Hangzhou 311300, China; yejunhua2020@zafu.edu.cn; 3 Zhejiang Key Laboratory of Intelligent Sensing and Robotics for Agriculture, Hangzhou 311300, China

**Keywords:** pig, tracking, re-identification, graph convolutional network, skeleton

## Abstract

Pig tracking contributes to the assessment of pig behaviour and health. However, pig tracking on real farms is very difficult. Owing to incomplete camera field of view (FOV), pigs frequently entering and exiting the camera FOV affect the tracking accuracy. To improve pig-tracking efficiency, we propose a pig-tracking method that is based on skeleton feature similarity, which we named GcnTrack. We used YOLOv7-Pose to extract pig skeleton key points and design a dual-tracking strategy. This strategy combines IOU matching and skeleton keypoint-based graph convolutional reidentification (Re-ID) algorithms to track pigs continuously, even when pigs return from outside the FOV. Three identical FOV sets of data that separately included long, medium, and short duration videos were used to test the model and verify its performance. The GcnTrack method achieved a Multiple Object Tracking Accuracy (MOTA) of 84.98% and an identification F1 Score (IDF1) of 82.22% for the first set of videos (short duration, 87 s to 220 s). The tracking precision was 74% for the second set of videos (medium duration, average 302 s). The pigs entered the scene 15.29 times on average, with an average of 6.28 identity switches (IDSs) per pig during the tracking experiments on the third batch set of videos (long duration, 14 min). In conclusion, our method contributes an accurate and reliable pig-tracking solution applied to scenarios with incomplete camera FOV.

## Simple Summary

Pig tracking has gradually become a requirement in the modern pork industry, facilitating the implementation of automated and intelligent management. Common cameras cannot monitor the entire pig housing in the production environment, which results in identity matching errors caused by pigs leaving and entering the camera’s detection area. This article proposed a pig-tracking method combined with re-identification. It provides technical support for automated and intelligent monitoring by enabling pig tracking in the modern pork industry.

## 1. Introduction

Real-time monitoring of the health status of pigs has gradually become important as pig farms develop in scale and intelligence. Monitoring the health status of pigs manually is often subjective and imprecise [1]. Traditional invasive pig monitoring methods based on RFID [2,3,4] suffer from issues of easy contamination, damage, and high cost [5]. With the development of computer vision and artificial intelligence technology, continuously monitoring pigs using videos is an effective solution for achieving automated pig management on pig farms. By continuously tracking the health status of pigs, managers can take timely measures when abnormalities occur.

Convolutional neural networks (CNNs), which are advanced artificial intelligence technologies, have been widely used in pig tracking. For example, Cowton et al. used the Faster R-CNN algorithm to detect pigs in videos and tracked them using the DeepSORT tracking algorithm [6]. Zhang et al. proposed a method that combines CNN detectors with filter-based trackers and designed a hierarchical data association algorithm that can accurately track pigs without manual labelling. This method addresses the tracking accuracy reduction problem caused by factors such as image deformation and the similar appearance of individual pigs [7]. Lu et al. developed a tracking method based on a rotating box detection model and designed a centre distance matching mechanism as an ID association method, which effectively reduced the detection failure problem caused by the high-speed movement of pigs [8]. Tu et al. proposed a multi-target tracking method that integrates YOLOv8 and OC-SORT, effectively improving tracking stability and behaviour monitoring accuracy in complex environments by optimizing trajectory processing and data association [9].

Although these CNN-based methods can track pigs effectively, their application relies on a common premise: the camera FOV must cover the entire pigsty, and a dataset with a full FOV for tracking is needed [8,9,10,11]. However, the recording equipment above pigsties on pig farms comprises mainly cameras, and the field of view (FOV) is usually the centre of the pigsties, which makes covering the entire pigsties difficult [12]. An incomplete camera FOV creates problems such as ID loss and discontinuous pig tracking during the tracking process, which reduce the tracking accuracy. The existing tracking methods that rely on the IOU and Kalman filtering cannot solve these problems. By combining tracking with reidentification (Re-ID) technology, the tracking limitations caused by the inability to track due to an incomplete camera FOV can be overcome to achieve longer and more accurate pig tracking.

Re-ID aims to identify and match the same individual from different camera perspectives [13]. In terms of feature representation, Re-ID methods can be classified into five categories [14]: global appearance methods [15,16], local part alignment methods [17], attention methods [18,19], transformer methods [20], and graph convolutional network (GCN) methods [21,22,23,24]. GCN is a specialised convolutional network that is used to process structured graph data. A GCN is used to handle complex relationships between entities to achieve more accurate Re-ID. The application of skeleton information to graph CNNs is a research hotspot because the connection relationships between skeleton keypoints, which constitute an innate adjacency graph representation, can reflect individual key information well. A GCN proposed by Ning et al. was used to encode the dependency relationships of human joints into a potential human posture representation, and the posture Re-ID problem was solved by calculating the encoding similarity [25]. Zhijun et al. effectively utilised human pose information to guide the network to focus on key regions and reassign local feature weights by combining a multibranch architecture, a pose-guided attention mechanism, and an adaptive graph attention module, which improved the person Re-ID accuracy [26].

Although Re-ID technology has been widely used in pedestrian detection, its application in daily pig monitoring has not been verified. The significant difference in structure between pigs and humans is a challenge, as is the similar hair colour and high degree of physical similarity among pigs. Additionally, poor lighting and limited space in the pigs’ living environment can also make Re-ID more difficult. Several researchers have marked pigs to reidentify them using the mark information. For example, Wang et al. tracked and Re-IDed weaned pigs by applying numerical identifiers to the animals and integrating the marks with deep learning strategies that utilise Re-ID attributes and the intersection over union (IOU) [27]. Guo et al. optimised the Re-ID association processes of JDE, FairMOT, and DeepSORT, reducing the number of ID exchanges [28]. These studies indicate that pig tracking via Re-ID technology is practical and feasible. Inspired by the application of Re-ID technology in human tracking, its application in pig tracking is practical and feasible, but existing related research is still lacking.

Here, we propose a novel method, GcnTrack, that utilises skeleton pose re-identification and aims to track pigs that reappear after disappearing, such as individuals who disappeared because they left the camera’s FOV, were occluded, or were missed by the detector. GcnTrack does not rely on labelling pigs or adding invasive sensors. Our study focuses mainly on pig tracking in scenes with an incomplete camera FOV. In this scenario, the pigs may leave the camera’s FOV frequently, move quickly, or block each other, which poses a challenge for tracking. Our research is based on the following theoretical basis. First, skeleton keypoints are highly interpretative because they are related to pig postures. Second, skeleton keypoints in consecutive frames have spatial consistency, which facilitates the inference of the similarity between two targets by calculating the feature similarity of the target keypoint coordinates in two successive frames. Finally, pig skeleton information is stable, which means that when pigs reappear after leaving the camera FOV for a short period, they can be Re-IDed through skeleton information.

## 2. Materials and Methods

### 2.1. Pigsty Environment

**Figure 1:**
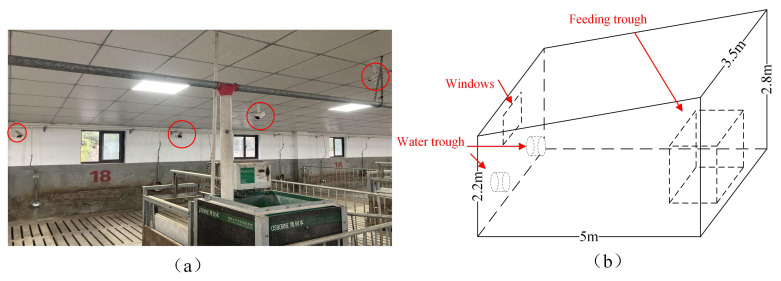
Video capture scene. (**a**) Camera position (**b**) Three-dimensional drawing of the piggery.

The videos used in this study are from the dataset established by Z. Wang et al. [29]. The video was recorded from July to October 2021. This dataset collected 14 Landrace pigs aged 60 days as experimental subjects (two pens, 7 pigs in each pen). Note that this dataset involved only routine activities and non-invasive video recording of pigs in a natural farm setting. No interventionist procedures were conducted. The actual scene of the pig house is shown in Figure 1. There are windows on the side of the pig house, which may be overexposed at noon. In addition to the activity area, there are waterers and feeding troughs on both sides of the pig house to facilitate capturing more postures. To obtain this dataset, the authors installed multiple-angle cameras on the ceiling of a pigsty to achieve multiview video capture of pigs from above. The captured video has 30 frames per second and a resolution of 1920 × 1080.

### 2.2. Data Collection and Processing

**Figure 2:**
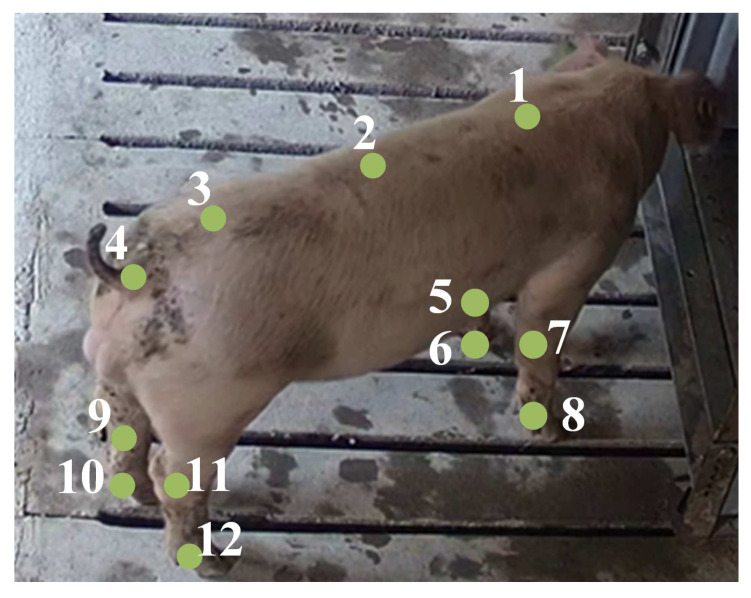
Pig skeleton keypoint connection labelling.

We selected the videos from the side view in the dataset from 7:00–17:00 as the effective data to ensure that the entire body of each pig was clearly captured. There were a total of 37 videos, each varying in length from 1 min to 14 min at 25 frames per second. We retained 6 videos as a tracking validation set. The annotated images in the original dataset, which were used only for skeleton keypoint detection and extraction, without incorporating time series, were not applicable to tracking and Re-ID. Therefore, we relabelled the keypoints of the pigs and their individual identities by retaining one frame per second. We mainly established the pig skeleton model based on the definition of pig joints in anatomy, and manually annotated the keypoints on the skeleton, as shown in Figure 2. Furthermore, for partially occluded pigs, manually inferring keypoint locations may introduce noise into the training data. Therefore, we only annotated their visible parts.

The skeleton model is divided into the upper body area and the lower body area, with a total of 12 key points. The upper body area has 4 points, including the main body area (tail, hip, spine centre point, and shoulder), and the lower body area has 8 points, representing the elbow and hoof joints of the limbs. We constructed a pig-tracking dataset with labels for training the pig target detection and tracking modules, which contained pig identity information and keypoint information such as the back, legs, and elbows for a total of 2020 images.

### 2.3. Dataset Establishment

**Figure 3:**
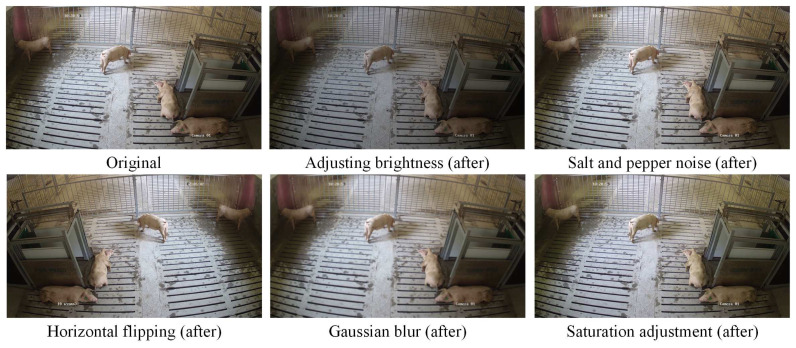
Examples of data enhancement.

We divided the keypoint detection dataset into training and testing sets at an 8:2 ratio and then augmented the images using methods such as salt and pepper noise addition, Gaussian blurring, brightness adjustment, saturation adjustment, and horizontal flipping, as shown in Figure 3.

All image processing was performed via Imgaug (v 0.4.0) and Random (Python version 3.7.0). We set the brightness to 0.5~1.5, the salt and pepper noise intensity to 0.01~0.05, the horizontal flipping probability to 0.5, the Gaussian blur to 0~0.8, and the saturation to −20~20. Two items were selected for each data enhancement. The number of seeds used was 42. After augmentation, the number of images in the dataset was increased from 2020 to 8080 to improve the model’s generalisability. We extracted the skeleton data of consecutive frames and obtained a total of 6513 pairs of data for training the GCN. For an extracted pair of skeleton keypoint sequences, they are defined as positive sample pairs if they originate from consecutive time points in the same pig, otherwise labelled as negative sample pairs. Containing 2742 positive pairs and 3771 negative pairs, the validation set and test set are divided in a 2:1 manner.

The pig-tracking validation set is labelled with pig ID information, as shown in Table 1. These videos originate from a different pigpen within the dataset and were not involved in model training; they have been uploaded to https://www.kaggle.com/datasets/hu233wu/pig-video (accessed on 9 February 2025). To evaluate the model’s precision in tracking and detecting pigs when they re-enter the camera’s FOV, we noted instances where pigs left the FOV throughout the videos. The number of such instances indicates the number of times the pigs moved from outside to inside the visible FOV. In the validation set, the pig skeleton keypoints in Videos 1 and 2 were specifically annotated to assess the accuracy of the model in extracting skeletons.

### 2.4. The Tracking Method Based on Skeleton Feature Similarity

**Figure 4:**
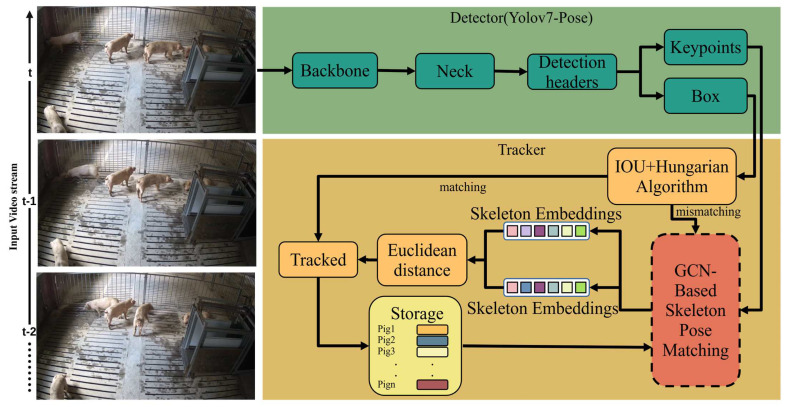
Flow chart of the pig detection and tracking algorithm. The tracker first uses the IOU for matching. If the match is successful, the algorithm terminates, and the match is stored. Otherwise, GcnTrack matches the current skeleton information with the skeleton information in storage through the Euclidean distance.

In this section, we detail our proposed pig-tracking method, GcnTrack, the general framework of which is shown in Figure 4. GcnTrack integrates YOLOv7-Pose, a GCN, and a Re-ID module that is innovatively designed based on skeleton pose matching and a dual-tracking strategy combined with the IOU for effective pig tracking. Specifically, YOLOv7-Pose is used to extract pig bounding boxes and keypoints. Then, the IOU and Hungarian algorithms are applied to associate pig IDs with the bounding boxes. The skeleton keypoints of pigs that are occluded or reappear after leaving the scene are input into the GCN for Re-ID to improve the tracking accuracy.

#### 2.4.1. Pig Skeleton Keypoint Detection

**Figure 5:**
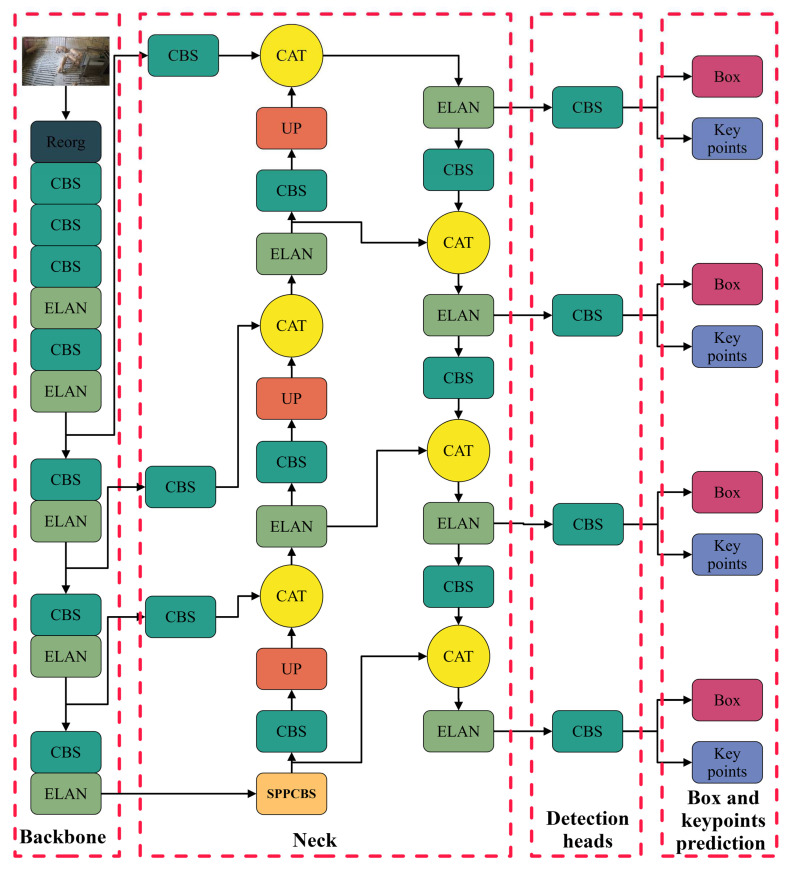
YOLOv7-Pose network structure.

Accurate pig skeleton keypoint extraction and bounding box detection are essential for accurate tracking. We utilised YOLOv7-Pose as the detector to extract the skeleton keypoints and bounding boxes of groups of pigs accurately; the network structure is illustrated in Figure 5. YOLOv7-Pose is an implementation of YOLOv7 [30], which is based on the YOLO-Pose algorithm, which is one of the most popular detection algorithms presently in use. YOLOv7-Pose associates human body keypoints using anchors to achieve joint detection of human targets and their keypoints. It uses the SPPCSPC module, E-ELAN module, and deep supervision technology to enrich the feature information and effectively alleviate the deep neural network training gradient disappearance and slow convergence problems.

YOLOv7-Pose has an extremely fast inference speed. Structural reparameterisation reduces the number of model parameters and improves the model’s operating efficiency.

#### 2.4.2. Dual-Tracking Strategy

Our tracking strategy tracks pigs on the basis of the target box and skeleton feature information detected by the detector combined with the GCN and the Hungarian algorithm. During the identity association phase of tracking, we focused on two complementary pieces of information: the consistency in the skeleton posture, and the consistency in the spatial information. The first association uses spatial consistency. If the IOU threshold between the bounding boxes for ${pig}_{i}$ exceeds 80% in successive frames k and k + 1, we consider the pig to be the same in both frames. This strategy is based on the assumption that the target’s movement from one frame to the next never exceeds a certain threshold. However, this assumption is not always reliable, especially when pigs are obscured, enter from outside the camera’s FOV, or move quickly. In this case, we need to match the reappearing pigs with unmatched candidate pigs. Previous studies have typically accomplished this task by analysing pig appearance characteristics. However, the pigs’ appearance features present significant differences at different camera angles, which may confuse the classifier and lead to incorrect recognition. Therefore, we introduced a secondary association on the basis of skeleton pose consistency, where GCN skeleton feature Re-ID is used to match IDs. The secondary association phase is designed to address instances where pigs reappear after an obstruction or move into the camera’s FOV from outside to determine whether these are new pigs or those that were previously lost to tracking.

A GCN is a methodology for applying CNNs to graphs with arbitrary structures. In a GCN, we represent data as a graph where the nodes represent entities such as pig skeleton keypoints and the edges represent the relationships or connections between these nodes, such as the linkages between keypoints in the skeleton. For a graph G = (V, E), where V represents the node set and E represents the edge set, the adjacency matrix A represents the graph’s structural connections, indicating the existence of connections between nodes i and j [31]. Traditional CNNs excel in processing grid-based data, such as images. In contrast, GCNs perform convolution operations on graph-structured data, learn node information, and retain the topological connectivity information between the nodes. GCNs are more suitable for solving graph-related problems [32].

**Figure 6:**
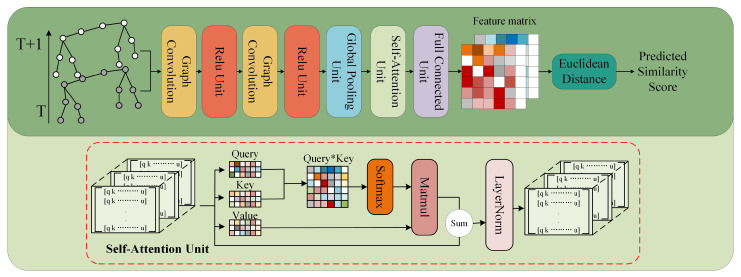
GCN network structure. The model extracts feature vectors from the input image and calculates the similarity between the vectors via the Euclidean distance.

Tracking pigs between successive frames can be viewed as a graph problem. Although the movement of pigs can cause changes in skeleton position, the amplitude of the skeleton changes between the two frames is minimal. This characteristic enables the use of a GCN to match skeleton postures. The keypoints of the pig skeleton are connected to form an adjacency matrix, feature vectors are extracted from the adjacency matrix between two frames using a GCN, and their similarity is calculated using cosine similarity. When the similarity between the keypoints of two pig skeletons exceeds 80%, we consider them to correspond to the same pig. Otherwise, we regard them as corresponding to different pigs. Referencing the Siamese network architecture proposed by Ning et al. [25], we developed a similar GCN module (Figure 6).

In the GCN framework, the contribution of each keypoint to the overall prediction task is uneven when processing pig skeleton keypoint data, and some keypoints play a more important role in Re-ID. We introduced a self-attention module to capture and utilise this inequality more effectively. The self-attention module can adaptively learn and emphasise keypoints that are more important for the current prediction task, enhance the focus of the model on important keypoints, and enable the entire network to perform more precise feature weighting and processing on a global scale. Self-attention achieves richer representation and finer-grained attention allocation by processing multiple attention heads in parallel. When pigs encounter occlusion, the self-attention mechanism effectively captures global dependencies. By leveraging visible keypoints information, it enhances the robustness of feature representation, thereby improving model accuracy.

In optimising Graph Convolutional Networks (GCNs), we enhanced the representational capabilities of the model by introducing Contrastive Loss, which aims to learn an effective embedding representation by optimising the similarity between samples [33]. The core idea of this loss function is to maximise the similarity between pairs of positive samples while minimising the similarity between pairs of negative samples, thus facilitating the model to effectively discriminate between different classes of samples. In this paper, the GCN input consists of a pair of samples, each represented as a graph of porcine skeleton data. Joint coordinates function as graph nodes, and skeleton connectivity information serves as graph edges. The model outputs two feature vectors. We assessed the similarity between feature vectors generated by GCN using Euclidean distance to determine whether samples originate from the same pig. To ensure that the feature vectors generated by the GCN are close enough for positive samples and far enough for negative samples, a specific contrast learning loss function is used for constraints. Its formula is as follows:

(1) $$L=\frac{1}{2N}\sum_{i=1}^{N}\left[y\cdot d^{2}+\left(1-y\right)\cdot max{\left(0,m-d\right)}^{2}\right]$$

where *d* denotes the Euclidean distance between pairs of samples, *m* is a predefined boundary value, *y* is the label (*y* = 1 for positive sample pairs and *y* = 0 for negative sample pairs), and *N* is the number of sample pairs.

The detailed computational framework is outlined in Algorithm 1.
**Algorithm 1:** Detailed calculation steps of the tracking algorithm**Assume**: now_keypoints stores the ID, bounding box, keypoint, and ID information of each pig in the fk; pre_keypoints stores the ID, bounding box, and keypoint information of the previous frame; the current set of lost pigs is referred to as lose_pig; now_pig_num is the total number of current pig IDs.; and max_pig_num is the maximum number of pigs. **Input:** now_keypoints, pre_keypoints, lose_pig, now_pig_num, max_pig_num 1: **for** frame fk in Video **do** 2:    **if** first_frame **then**: 3:     label.init(keypoints) 4:    **end** 5:    iou = calculate_iou_match(now_keypoints, pre_keypoints, cost_limit = 0.3) 6:    **if** all pigs are successfully matched **then**: 7:     lose_pig = update(now_keypoints, pre_keypoints) 8:     update(label) 9:    **end** 10:    similarity=calculate_gcn _match(keypoints, lose_pig, cost_limit=0.2) 11:    **if** all pigs are successfully matched **then**: 12:     lose_pig = update(now_keypoints, pre_keypoints) 13:     update(label) 14:    **end** 15:   **if** now_pig_num < max_pig_num **then**: 16:     now_pig_num+1 17:     lose_pig = update(now_keypoints, pre_keypoints) 18:     update(label) 19:    **end** 20:    similarity=calculate_gcn_match(keypoints, lose_pig) 21:    lose_pig = update(now_keypoints, pre_keypoints) 22:    update(label) 23: **end** **Output**: label

## 3. Results

### 3.1. Experimental Setup

#### 3.1.1. Experimental Platform and Parameter Settings

All the experiments in this study were conducted on the same computer which ran Ubuntu 23.04 operating system, and was equipped with an Intel i7-13700KF CPU and an NVIDIA GeForce RTX 3090Ti graphics card. The software environment included Python 3.7.0 and PyTorch 1.13.1. The YOLOv7-Pose model was trained on the pretrained weights specified by C.-Y. Wang et al. [30], and we trained for 200 epochs on our dataset. The batch size was 32, and the learning rate was 0.01. To ensure the generalization of the model, during the YOLOv7-Pose model training process, it was responsible only for detecting pigs and extracting pig skeletons; ID classification was not required. The GCN used the skeleton information extracted from our tracking dataset for training. The training dataset comprised 8786 pieces of data, whereas the testing dataset comprised 4240 pieces of data. The GCN was trained for 300 epochs using the Adam optimiser, with an initial learning rate set of 0.001 and a learning rate reduction of 0.0001 every 50 epochs, and the batch size was set to 64. We set the IOU threshold to 0.3 during the first association and the Euclidean distance threshold to 0.2 during the second association.

#### 3.1.2. Evaluation Metrics

MOTA comprehensively considers missed detections, false detections, and identity changes and is often used to measure the overall performance of tracking algorithms. MOTA is defined as follows:

(2) $$\mathrm{MOTA}=1-\;\frac{\mathrm{FN}+\mathrm{FP}+\mathrm{IDSW}}{\mathrm{GT}}\;$$

where IDSW (ID switches) represents the number of times that labels are changed for objects tracked by the MOT algorithm; FN represents the total number of missed detections in the entire video, which is the total number of missed detections of pigs; FP is the number of incorrectly detected targets, which is the total number of non-pig targets detected as pigs; and GT signifies the total number of pigs that are truly present in each frame.

We used IDF1 (Equation (5)) to evaluate the tracker’s ability to maintain target IDs over multiple frames. IDF1 focuses on the target identity consistency of the tracking algorithm, aiming to evaluate the ability of the target to maintain the correct identity for a long time in the tracking algorithm. Specifically, the precision (IDP) (Equation (3)) gauges the precision of the ID of each detected pig, and the recall (IDR) (Equation (4)) represents the recall rate for the pig IDs.

(3) $$\mathrm{IDP}=\frac{\mathrm{IDTP}}{\mathrm{IDTP}+\mathrm{IDFP}}$$

(4) $$\mathrm{IDR}=\frac{\mathrm{IDTP}}{\mathrm{IDTP}+\mathrm{IDFN}}$$

(5) $$\mathrm{IDF}1=\frac{2*\mathrm{IDP}*\mathrm{IDR}}{\mathrm{IDP}+\mathrm{IDR}}=\frac{2*\mathrm{IDTP}}{2*\mathrm{IDTP}+\mathrm{IDFP}+\mathrm{IDFN}}$$

where IDTP is the number of times the model detects objects correctly, IDFP is the number of times the model detects objects incorrectly, and IDFN is the number of times the model misses detections.

We used mostly tracked (MT) and mostly lost (ML) to measure the performance of the tracker throughout the video. A pig is considered MT if it is tracked for more than 80% of the video and ML if it remains untracked for the same threshold.

We used OKS to assess the skeleton keypoint extraction ability of the model. OKS provides a method for quantifying the accuracy between predicted keypoints and actual keypoints, accounting for the keypoint position accuracy and visibility. OKS is calculated as follows:

(6) $$\mathrm{OKS}=\frac{\Sigma_{i}\exp(-{d_{i}}^{2}/\;2{s\;}^{2}*{k_{i}}^{2})\;*\;\delta (v_{i}>\;0)\;}{(\Sigma_{i}\;[\delta (v_{i}\;>\;0)])}$$

where $d_{i}$ represents the Euclidean distance between the predicted and actual keypoint i, and *s* indicates the scale of the pigs. The constant $k_{i}$ controls the importance of keypoint i, $v_{i}$ represents the visibility label of keypoint i, and δ is defined as an indicator function. AP50, AP75, and AP90 represent average precision at OKS thresholds of 0.50, 0.75, and 0.90, respectively.

The accuracy of the GCN is evaluated using Accuracy, as defined by the following formula:

(7) $$Accuracy=\frac{TP+TN}{TP+TN+FP+FN}$$

Here, TP denotes true positive matches, TN represents true negative matches, FP refers to false positive matches, and FN indicates false negative matches.

### 3.2. Keypoint Detection Evaluation

The results in Table 2 indicate that YOLOv7-Pose performed well in the OKS evaluation, demonstrating its efficiency and accuracy in keypoint detection. In particular, in scenarios involving partially visible keypoints, YOLOv7-Pose displayed robustness and adaptability. YOLOv7-Pose outperformed DeepLabCut [34] and AlphaPose [35] in the high threshold category, especially with the AP75 reaching 0.78, surpassing DeepLabCut’s 0.49 and OpenPose’s 0.67. These results indicate that the YOLOv7-Pose algorithm has higher detection accuracy for keypoints in pig skeletons under high threshold conditions.

**Figure 7:**
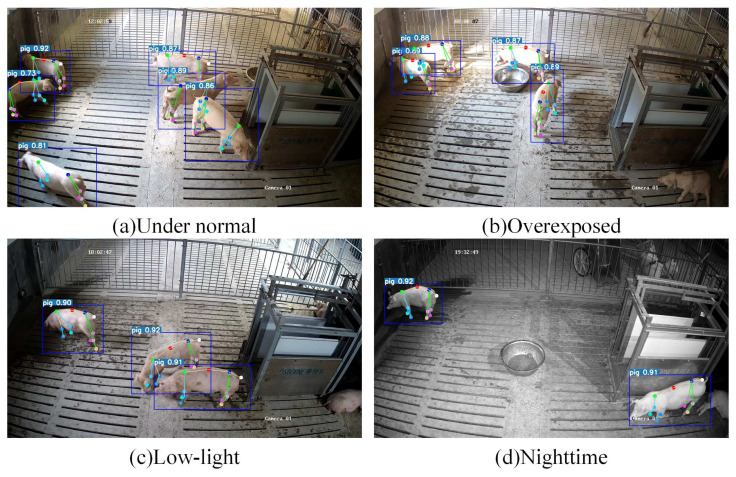
Detector results under different lighting conditions.

Figure 7 presents example detection results of the detector under varying lighting conditions; Yolov7-Pose successfully identifies pigs under normal, low-light, overexposed, and nighttime conditions. In most instances, YOLOv7-Pose exhibits superior detection performance and accurately extracts keypoints, with only a few pigs showing instances of keypoint loss or positional deviation. We trained the model with augmented data, enhancing its robustness and allowing it to maintain excellent detection performance under complex lighting conditions like dim light and overexposure.

### 3.3. Tracking Effect Evaluation

#### 3.3.1. Comparison of Different Tracking Approaches

To evaluate the effectiveness of the dual-tracking strategy, we compared it with using only a GCN for skeleton pose matching tracking and the ByteTrack [36] tracking algorithm and the BotTrack [37] tracking algorithm. The results are shown in Table 3.

According to Table 3, compared with the dual-tracking strategy, ByteTrack and BotTrack have significant differences in terms of indicators such as MOTA and IDF1. The main reason is that they cannot effectively solve the pig reidentification problem. When a pig reappears after being occluded or temporarily leaving the scene, these algorithms usually assign a new ID, resulting in a higher IDS and lower values of the other indicators; thus, they perform poorly in scenes with incomplete perspectives. BotTrack’s tracking performance shows negligible improvement when Re-ID using appearance features is enabled. This is likely due to the similar coat colour and appearance of pigs, making it difficult to distinguish them based on appearance alone. The dual-tracking strategy employs skeleton keypoints for tracking, demonstrating a significant advantage in Re-ID accuracy over the BotTrack method, which relies on appearance features. The dual-tracking strategy utilizing GCN effectively addresses the Re-ID challenges for pigs obscured during occlusion or those re-entering the FOV after departure, thereby enhancing tracking performance.

#### 3.3.2. Validation of Tracking Results

**Figure 8:**
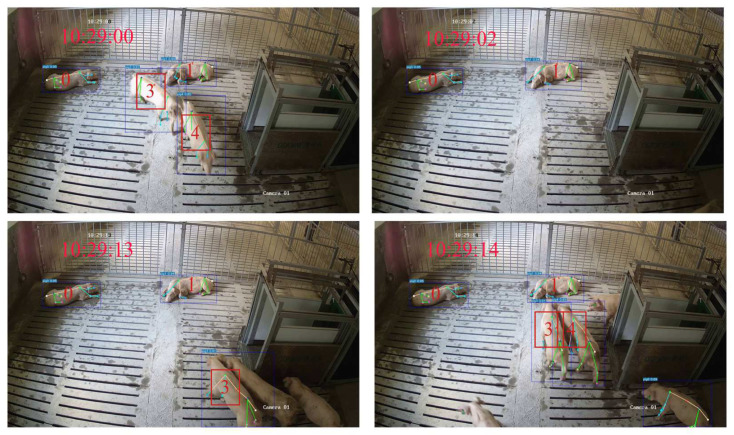
Example of GcnTrack’s successful reidentification when pigs left the camera FOV. Pig 3 and Pig 4 left the camera FOV at 10:29:02. Although they were gone for approximately 11 s, GcnTrack still correctly assigned the original IDs.

**Figure 9:**
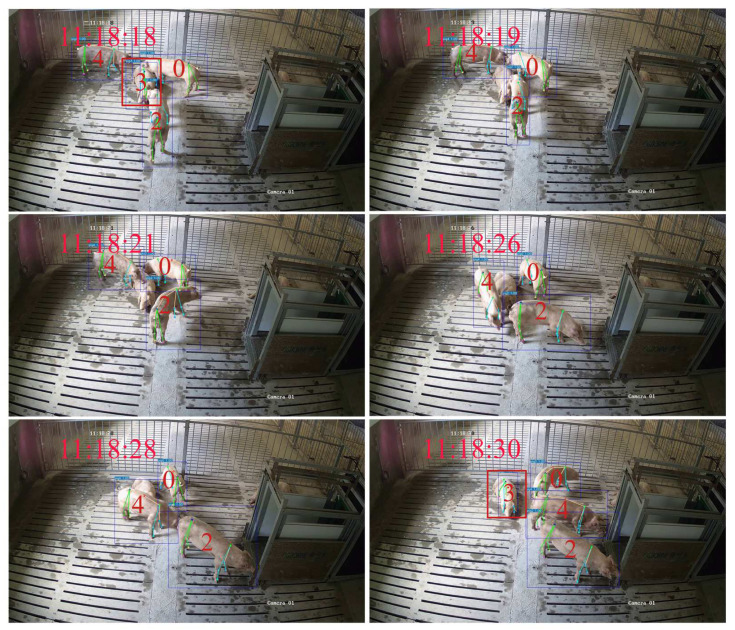
Example of GcnTrack successfully reidentifying occluded pigs. Pig 3 was occluded at 11:18:19 and reappeared in the camera’s FOV until approximately 11:18:30, and GcnTrack reidentified it correctly.

A frequent scenario in this dataset involves pigs entering and exiting the camera’s FOV. Taking Figure 8 as an example, when pigs 3 and 4 leave the camera’s FOV for approximately 11 s, GcnTrack can still successfully perform Re-ID on the basis of the skeleton information. Another common situation is that pigs obstruct each other, causing the detector to miss detections. Pig 3 was successfully IDed and tracked after being severely obstructed for approximately 12 s, as shown in Figure 9. In such scenarios, ByteTrack and BotTrack suffer from ID replacement due to pig loss, whereas our method benefits from the Re-ID module and is almost unaffected.

**Figure 10:**
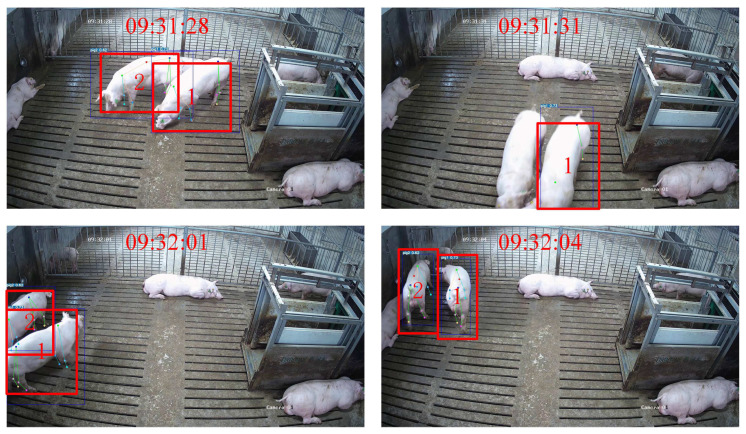
Examples of tracking pigs at different ages. Pigs 1 and 2 can still be re-identified approximately 30 s after leaving the area.

We evaluated the tracking performance of GcnTrack on pigs at various growth stages using Video 7. As shown in Table 3, compared to other videos, MOTA significantly decreased while IDF1 exhibited minor variation. Additionally, the Individual Detection Percentage dropped by 0.15, and IDS increased noticeably. The analysis indicates a decline in detector performance, likely attributed to the rapid and significant appearance changes in pigs within a short 60-day growth cycle. This reduction in detection accuracy indirectly affected the tracking performance of GcnTrack. As illustrated in Figure 10, the detector missed some pigs, and there were inaccuracies in certain keypoints. This led to a rise in IDS and a drop in MOTA, impacting GcnTrack’s overall tracking performance.

We utilised Video 8 to evaluate the model’s tracking performance in low-light conditions. As indicated by the results in Table 3, metrics such as MOTA and IDF1 show a slight decline compared to normal lighting conditions. This may be attributed to a decrease in detector performance, which indirectly affects the tracker’s efficacy. This indicates that variations in lighting and changes in pig appearance can influence the detector’s effectiveness, thereby affecting the tracker’s performance.

### 3.4. Ablation Study

#### 3.4.1. Ablation Study of Tracking Methods

We conducted ablation experiments using both the dual-tracking strategy and the GCN-only strategy. Since IOU combined with the Hungarian algorithm lacks association capability, it was excluded from the comparison. Table 4 also shows that the dual-tracking strategy is more effective than the GCN-only strategy and that the MOTA, IDF1, MT, and accuracy rates are all improved, which is mainly because the dual-tracking strategy combines IOU matching, which reduces the effects of missed key point detections and false detections on the model. Reliance only on the GCN network is more prone to errors than the dual-tracking strategy is. The results in Table 4 also indirectly confirm our viewpoint that our dual-tracking strategy reduces the IDS (average number of pigs switching IDs) compared with tracking with the GCN tracking module alone. The individual-tracking percentage increased from 0.67 to 0.73, confirming the effectiveness of our proposed algorithm for tracking pigs.

#### 3.4.2. Ablation Study of GCN Depth

We also compared the impact of GCN depth on tracking performance. The data in Table 5 reveal that the GcnTrack tracking module had little impact on the original network model’s FPS, maintaining a small gap from the YOLOv7-Pose network’s baseline inference speed of 23.75 FPS/s. This is because GCNs have a fast inference speed, and the Hungarian algorithm has extremely low performance overhead when solving assignment problems. In addition, for shorter videos, the two-layer GCN model performed well in terms of metrics such as model precision, MOTA, IDF1, and tracking precision. The accuracy of the four-layer model showed minimal decline; however, tracking precision decreased by 0.09 compared to the two-layer model. This indicates that deeper models are more prone to overfitting and exhibit poorer generalization. This may be due to deeper networks being more susceptible to over-fitting during feature propagation, causing keypoint features to converge and become indistinguishable. Therefore, we ultimately used a two-layer GCN.

#### 3.4.3. Ablation Study of Keypoints

To assess the impact of keypoints on GCN, we conducted a keypoint ablation study using the validation set. Keypoints in the validation set are set to (0, 0) based on individual keypoints, connectivity, and categories, and the results are shown in Table 6. Table 6 only presents results with Accuracy changes below 0.1.

Results in Table 6 demonstrate significant variations in the impact of missing keypoints on the performance of the GCN model. The loss of hoof joints has the most pronounced effect on the model, with the absence of four hoof joints resulting in a 2.6% decrease in accuracy. The absence of back keypoints has a minimal effect on the model, with the loss of all four back keypoints resulting in only a 0.16% decrease in accuracy. This is attributed to the significant dynamic changes exhibited by hoof joints during movement, whereas back keypoints display minimal variation, making them harder to distinguish. Consequently, the model is more sensitive to changes in hoof joints.

The loss of individual keypoints has minimal impact on the model, not exceeding 0.1%. This is mainly because our training dataset already includes instances with missing keypoints. The model exhibits a degree of robustness as it has been exposed to data with missing keypoints during training. Moreover, when an individual keypoint is missing, the characteristics of GCNs allow them to compensate by extracting information from neighbouring keypoints, thereby mitigating the impact of missing keypoints on the model.

### 3.5. Tracking Test on a Long Video

We tested the performance of GcnTrack using a dual-tracking strategy and a two-layer GCN on Video 6 in our validation set. Table 7 shows the tracking results for each pig in the video. The calculation of the individual-tracking percentage is based on the cumulative number of frames during which the pigs maintain their correct ID, explicitly excluding frames after the ID changes. In Video 6, the average IDS for the pigs was 6.28, with an average re-entry FOV of 15.29. These results indicate that GcnTrack can correctly reidentify and maintain tracking coherence most of the time.

**Figure 11:**
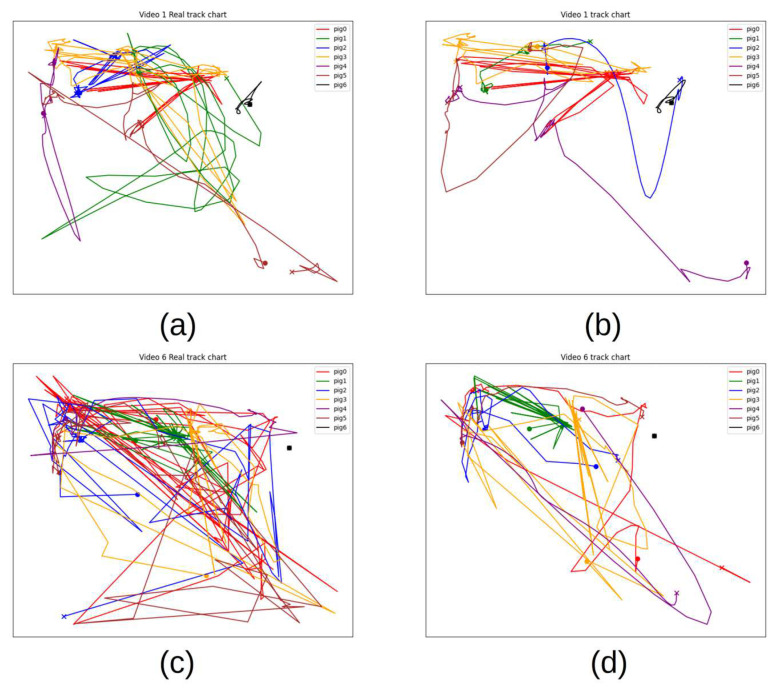
Tracking trajectories: (**a**,**b**) both come from Video 1, and (**c**,**d**) both come from Video 6. The letter “o” indicates the beginning of tracking, and “x” indicates the end. The plots on the left show the actual trajectories, and the plots on the right show the tracking trajectories.

Figure 11 displays actual trajectory and tracking trajectory plots for each pig in Videos 1 and 6. These paths were plotted at one-second intervals and smoothed using a Bezier curve. In pig farms, the dense activities, frequent occlusions, and irregular movement patterns of pigs pose great challenges to the tracking algorithm. The trajectories of some pigs, such as pig 2 in Figure 11b, are incorrectly truncated. By observing the video, it was found that pig 2 was repeatedly blocked by other pigs within a short period in the venue, which eventually led to the incorrect update of its ID. When analysing the actual tracking trajectory images labelled a and c, we observed that the pigs moved more frequently in the upper-left corner and the middle-right area. These areas were identified as the locations of the water and food troughs. These results indicate that these areas were highly attractive to pigs, presumably because their basic physiological needs, such as drinking and feeding, were met in these areas.

## 4. Discussion

To capture video data for most existing pig-tracking algorithms, cameras are placed directly above the pigsty for tracking from a fully covered perspective. However, in real pig farms, the pigsty height is insufficient for the camera FOV to cover the entire pigsty. For example, in our dataset, the height of the pigsty is 2.8 m, which is not sufficient to support a camera FOV that covers the entire pigsty. In previous studies, Wang et al. [27] considered the issue of pigs frequently entering and exiting the FOV and changing their IDs due to the difficulty of fully covering the pigsty with the camera’s view. After the pigs’ bodies were labelled, a model was used to extract visual features, which were then combined with the IOU metric to facilitate tracking. However, owing to the use of visual markers to assist in identifying pigs, this method may not be generalisable and cannot be applied to new pigs. Our approach extracts pig skeleton features and combines them with the IOU for tracking, effectively addressing the issue of pig ID changes caused by pigs frequently entering and exiting the FOV. Without relying on specific pig characteristics, the method can also be applied to new pigs.

Our application of a GCN in pig tracking is not the first such application. For example, Parmiggiani et al. [38] created a pig-tracking model based on a GCN. Each pig across successive frames is viewed as a network node in this method. The results of neighbouring frames are connected by constructing a bipartite graph. The advantage of this method is that by connecting nodes across multiple frames, the model can redetect and restore the original identities of the pigs lost in the previous few frames if the graph is sufficiently large. Unlike the method presented in this article, each pig is considered a node in the author’s approach, and every 25 frames, the nodes are connected to form an entire graph, which is then processed. However, this method can process data from only the previous 25 frames and is not capable of real-time tracking. Our method treats each pig skeleton’s information as a whole graph and uses a GCN to analyse the differences between the graphs of different pigs to track the pigs.

In terms of time consumption, as video duration and target quantity increase, the overall computational cost experiences a slight rise. For example, with 10 pigs, in the extreme scenario where all are lost, the Re-ID module must be invoked for matching when a new pig appears. The GCN must run 10 times, totalling about 0.01 s, making the overall computation-al burden almost negligible. As a result, the tracking module in GcnTrack has almost no impact on the detector, and the inference speed depends on the inference speed of YOLOv7-Pose. Our module can be inserted into any model that supports object detection and skeleton extraction with almost no performance loss.

**Figure 12:**
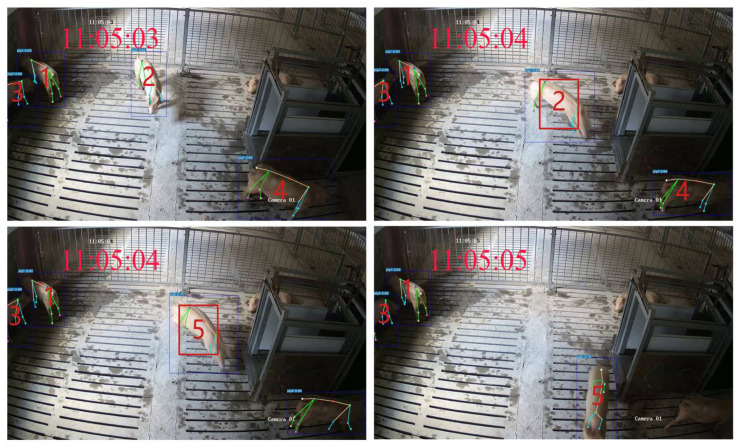
Tracking error example.

In GcnTrack, a GCN is used as the Re-ID module, which makes the performance of GcnTrack much better than that of ByteTrack and BotTrack in scenarios where pigs are obscured or leave the FOV and then re-enter. Although GcnTrack shows improved tracking performance in scenes with incomplete camera FOVs, it still cannot completely solve the problem of ID misconversion, which is a fundamental challenge that needs to be addressed in MOT. In the future, we can further improve the algorithm’s Re-ID capabilities by considering other identity features of pigs, such as gait, pig face, body shape, and coat colour. We tried to analyse the cause of the ID error. One reason is the rapid movement of the pigs, which exceeds the tracker’s threshold, directly causing ID mismatches. Taking Figure 12 as an example, Pig 4 moves quickly between the two frames, causing the ID to change. Another reason is errors in the detector’s results, such as a high number of false positives and missed keypoints, which severely affect tracking accuracy. Therefore, the results of the detector affect the tracking performance of the model.

Illumination variations can affect detector outcomes to some extent, which in turn influences MOT performance. Our detector is trained on augmented data, ensuring a certain level of robustness and maintaining good performance in overexposed and low-light conditions. In actual scenes, limited by lighting conditions, the detector experiences some performance degradation, leading to increased missed and false keypoints, which weakens tracking effectiveness. Future improvements could involve incorporating more data from low-light and nighttime scenarios to enhance the model’s robustness.

Long-term tracking is also a challenge in MOT. However, long-term tracking methods based on computer vision do not perform well [39]. Fattening pigs grow rapidly, and their appearance characteristics change significantly, which causes more missed detections and false detections by the detector, thereby weakening the tracking performance of GcnTrack. Because GcnTrack does not use specific features to identify pigs, our algorithm cannot correct ID errors once they occur, resulting in unsatisfactory tracking performance in long-term videos. To solve this problem, a detector with identity recognition can be introduced to regularly correct pig IDs to improve tracking.

GcnTrack focuses on tracking pigs in scenarios where the camera’s FOV is incomplete and should be applicable to other animals that resemble pigs. This tracking method, which is based on skeleton feature similarity, does not rely on the specific individual features of pigs. This study was a preliminary exploration of the use of tracking algorithms in actual farm applications, and the proposed method outperformed the existing methods for pig tracking. However, many issues remain to be resolved in future research, such as correcting incorrect IDs in long-term tracking and overcoming the appearance changes caused by the rapid growth of fattening pigs and light changes in pig houses. The rapid growth cycle and significant appearance changes in pigs can severely impact detector performance, potentially leading to its gradual failure. One potential research direction is developing an all-stage pig object detector. By incorporating datasets of pigs at various ages, a generalized detection model can be trained to enhance robustness and adaptability across different growth stages. Another possible research direction involves leveraging relatively stable features such as pig facial characteristics for tracking. This approach enables the model to continuously correct errors during the tracking process, thereby achieving long-term stable tracking of individual pigs. Long-term tracking is beneficial for continuous and automatic monitoring of pig health. We will consider long-term tracking of individual pigs in the future and introduce a detector with identity recognition to regularly correct pig IDs to improve tracking. We hope to use more datasets and parameters to track pigs and monitor the condition of individual pigs.

## 5. Conclusions

In this paper, we propose GcnTrack, which is a method for pig tracking using computer vision under limited-FOV conditions. GcnTrack utilizes Yolov7-Pose as the detector, employing IOU and the Hungarian algorithm for pig tracking, while leveraging GCN for trajectory association of lost targets. The strength of the GcnTrack method lies in its Re-ID capability, which utilizes pig skeleton feature information to re-identify pigs that leave and re-enter the camera’s FOV, significantly enhancing tracking accuracy in scenarios with incomplete camera coverage. The GcnTrack achieved a precision rate exceeding 74% for both short and medium length videos and a tracking accuracy of 48.29% for long videos, significantly outperforming methods such as ByteTrack and BotTrack. In summary, GcnTrack can effectively handle ID conversion when pigs frequently leave the FOV during the tracking process. It performs well for tracking pigs in long, medium, and short videos and provides an effective pig-tracking solution for pig farms.

## Figures and Tables

**Table 1:** Information on the videos in the validation set.

Video Number	Duration (s)	Number of Pigs	Number of Times that Pigs Left	Recording Date
Video 1	90	7	14	7.14
Video 2	220	7	10	7.15
Video 3	87	7	8	7.15
Video 4	302	7	16	7.21
Video 5	302	7	14	7.16
Video 6	842	7	35	7.14
Video 7	302	7	12	9.08
Video 8	302	7	14	7.15

**Table 2:** Comparison of key point extraction methods. All results are measured based on OKS.

Method	Backbone	AP50	AP75	AP90
DeepLabCut [34]	ResNet	0.84	0.49	0.36
AlphaPose [35]	ResNet	0.88	0.67	0.21
YOLOv7-Pose	YOLO	0.87	0.78	0.36

**Table 3:** Comparison of the tracking performance of different tracking methods in terms of the MOT evaluation metrics (for Videos 1, 2, 3, 4, 5, and 7 from the validation dataset).

Method	Videos	MOTA	IDF1	MT	ML	Tracking Precision	Individual Detection Percentage	Individual Tracking Percentage	IDS
ByteTrack [36]	1, 2, 3	25.87	24.70	0.30	0.16	0.23	0.87	0.19	22.57
	4, 5	29.15	29.74	0.27	0.1	0.22		0.14	39.14
BotTrack [37]	1, 2, 3	26.06	28.70	0.36	0.09	0.21	0.87	0.21	24.65
	4, 5	30.64	24.64	0.27	0.1	0.25		0.17	37.66
BotTrack(Re-ID) [37]	1, 2, 3	26.34	28.40	0.36	0.09	0.21	0.87	0.20	22.42
	4, 5	29.91	25.64	0.27	0.1	0.26		0.19	34.65
Dual-tracking strategy (ours)	1, 2, 3	84.98	82.22	0.79	0	0.83	0.87	0.80	1.61
	4, 5	82.36	76.00	0.85	0	0.74		0.73	2.35
	7	59.65	70.15	0.57	0	0.65	0.72	0.60	4.76
	8	79.32	73.76	0.71	0	0.70	0.83	0.69	2.60

**Table 4:** Results of Ablation Study.

Method	Videos	MOTA	IDF1	MT	ML	Tracking Precision	Individual Detection Percentage	Individual Tracking Percentage	IDS
Only GCN strategy	1, 2, 3	84.15	77.36	0.75	0	0.70	0.87	0.75	2.00
	4, 5	81.34	69.78	0.85	0	0.61		0.67	2.36
Dual-tracking strategy	1, 2, 3	84.98	82.22	0.79	0	0.83	0.87	0.80	1.61
	4, 5	82.36	76.00	0.85	0	0.74		0.73	2.35

**Table 5:** Comparison of the tracking performance of GCN networks with different numbers of layers (on Videos 1, 2, 3, 4, and 5 from the validation dataset) in terms of the MOT metrics.

Gcn Depth	Videos	Model Accuracy	MOTA	IDF1	MT	ML	Tracking Precision	Individual Tracking Percentage	IDS	FPS
One Layer	1, 2, 3	88.72	84.67	81.53	0.80	0	0.79	0.74	1.62	23.51
	4, 5		81.00	55.50	0.85	0	0.43	0.62	2.5	
Two Layers	1, 2, 3	89.46	84.98	82.22	0.79	0	0.83	0.80	1.61	23.51
	4, 5		82.36	76.00	0.85	0	0.74	0.73	2.35	
Three Layers	1, 2, 3	88.88	81.79	81.25	0.93	0	0.76	0.77	2.04	23.50
	4, 5		81.51	72.00	0.85	0	0.63	0.66	2.28	
Four Layers	1, 2, 3	89.06	81.33	80.33	0.93	0	0.74	0.71	1.91	23.45
	4, 5		81.56	71.47	0.92	0	0.64	0.69	2.35	

**Table 6:** Ablation study of keypoints.

Keypoints	Accuracy	Accuracy Change
-	89.46	-
hoof joints (6, 8, 10, 12)	87.14	−2.32
elbow joints (5, 7, 9, 11)	87.83	−1.63
hoof joints (6, 10)	88.81	−0.65
hoof joints (6, 8)	88.99	−0.47
elbow joints (5, 9)	89.12	−0.34
back keypoints (1, 2, 3, 4)	89.25	−0.21
back keypoints (1, 4)	89.30	−0.16
elbow joints (5, 11)	89.33	−0.13

**Table 7:** Tracking results for each pig in Video 6.

ID	Total Number of Frames ^1^	Individual Tracking Percentage	IDS	Number of Times the Pig Disappears ^2^	Detection Precision
0	4134	55.35%	14	28	92.44
1	10,946	11.86%	9	23	82.39
2	11,258	73.44%	2	17	99.08
3	7540	6.55%	11	20	96.67
4	2106	98.77%	0	5	98.78
5	4914	15.87%	7	13	94.50
6	1092	76.19%	1	1	93.33

^1^ Total frame count featuring pigs in the video; ^2^ Number of individual disappearances, including those due to missed detections and occlusions.

## Data Availability

The validation set used in this study can be obtained at https://www.kaggle.com/datasets/hu233wu/pig-video (accessed on 9 February 2025). The research data is available when required.

## Abbreviations

The following abbreviations are used in this manuscript:

FOV	Field of view
OKS	Object keypoint similarity
CNN	Convolution neural network
GCN	Graph convolutional network
Re-ID	Reidentification
IDF1	Identification F1 score
MOTA	Multiple object tracking accuracy
IOU	Intersection over union
IDS	Identity switch
MT	Mostly tracked
ML	Mostly lost
AP	Average precision
FPS	Frames per second
MOT	Multiple object tracking
Box	Bounding box
